# Tumor of Labium Majus

**Published:** 1916-08

**Authors:** 


**Tumor of Labium Majus.** J. W. Crawshaw, New Zealand Med. Jour., April. Patient aged 48. 8 years ago, after birth of last child, noticed a fleshy wart. For four years, it grew very slowly and caused no inconvenience but it then developed a pedicle and grew more rapidly, interfering with walking. The week after menstruation ceased, it increased in size, shrinking after another week. The dimensions were: length 5 inches, greatest diameter 3/16, pedicle, 1/2 inch long, greatest diameter below pedicle about 1 inch. He quotes Howard Kelly as having been able to collect only 20 cases.

